# Evaluation of the Antibacterial Activity of Acetic Acid in Comparison With Three Disinfectants Against Bacteria Isolated From Hospital High-Touch Surfaces

**DOI:** 10.1155/sci5/7598027

**Published:** 2025-03-26

**Authors:** Ayesha Muazzam, Sidrah Saleem, Hafiz Muhammad Faizan Nadem, Faiz Ul Haq, Ghaniya Ali, Nida Javed

**Affiliations:** ^1^Department of Microbiology, University of Health Sciences, Lahore, Pakistan; ^2^Department of Nuclear Medicine, Pakistan Kidney and Liver Institute & Research Center, Lahore, Pakistan; ^3^Department of Pathology, Al Aleem Medical College Lahore, Lahore, Pakistan

**Keywords:** acetic acid, bacterial pathogens, disinfectants, high-touch surfaces, hospital infection, phenol

## Abstract

Acetic acid, a readily available and less toxic alternative to conventional disinfectants, is widely used for cleaning in household settings. This study evaluates the antibacterial efficacy of acetic acid against bacteria isolated from hospital high-touch surfaces, comparing its performance to commonly used disinfectants, including phenol, sodium hypochlorite, and didecyldimethylammonium chloride (DDAC). A total of 120 samples were collected from high-touch surfaces in specialized patient areas. The antibacterial activity of acetic acid, phenol, sodium hypochlorite, and DDAC was assessed using the standard broth microdilution method against the isolated bacterial strains. From the 120 samples, 140 bacterial isolates were obtained. Acetic acid demonstrated strong antibacterial activity, with mean minimum inhibitory concentrations (MICs) ranging from 0.05 ± 0.00 to 0.25 ± 0.06 μL/mL, effectively inhibiting coagulase-negative *Staphylococcus* (CONS), *Klebsiella pneumoniae*, *Proteus vulgaris*, *Enterococcus* species, and *Serratia marcescens*. Its performance surpassed phenol and DDAC against these strains. Phenol exhibited higher MICs (0.50 ± 0.00 to 0.83 ± 0.10 μL/mL), indicating lower efficacy, while DDAC (0.06 ± 0.00 to 0.17 ± 0.04 μL/mL) and sodium hypochlorite (0.06 ± 0.00 to 0.10 ± 0.00 μL/mL) demonstrated comparable antibacterial effects. Phenol and sodium hypochlorite were found nonsignificant, while DDAC is highly effective at a concentration of 8.5%. Hospital surfaces were found to be contaminated with diverse bacterial strains. Acetic acid demonstrated significant antibacterial efficacy against both gram-positive and gram-negative bacteria, with MICs ranging from 0.05 ± 0.00 to 0.25 ± 0.06 μL/mL, suggesting its potential as an effective, economical, and less toxic alternative to conventional disinfectants.

## 1. Introduction

Hospital-acquired infections (HAIs) occur during the hospital stays and a vast variety of pathogens like multi–drug-resistant (MDR) bacteria including vancomycin-resistant *enterococci* (VRE), *Clostridium difficile*, *Pseudomonas aeruginosa*, *Acinetobacter spp*., and methicillin-resistant *Staphylococcus aureus* (MRSA) are often isolated from the hospital environment. These microorganisms continue to exist on hospital environmental surfaces, where they may serve as a source of infections [1].

Since HAIs are a leading cause of morbidity and mortality worldwide, surface contamination with microbes in particular is a public health concern [2]. The European Centre for Disease Prevention and Control (ECDC) found that the majority of the 33,000 persons dying each year in European countries secondary to infections caused by MDR bacteria were transmitted through hospitals [3]. Hospital environmental contamination should be considered as an integrated strategy for infection prevention and control. HAIs prolong hospital stay for the admitted patients, where they further continue to contaminate the inanimate surroundings [4]. It was observed that how the growth of enterohemorrhagic *E. coli* (EHEC) 0157:H7 was affected by the bacteriostatic and bactericidal effects of vinegar in food materials. A 0.1% quantity of acetic acid in the vinegar reduced the proliferation of all isolates examined [5]. The important aim of the current study is to assess the antibacterial properties of acetic acid against bacteria isolated from hospital surfaces in comparison with phenol, sodium hypochlorite, and quaternary ammonium compound (QAC).

In hospitals and other healthcare facilities, biocides (mainly antiseptics and disinfectants) have been widely utilized. It is used to disinfect a variety of medical instruments and to lessen the bioburden in the surroundings. Disinfectants, in particular, are crucial for infection control measures and the reduction of the spread of pathogenic microorganisms [6].

A common infection control approach is the widespread application of chemical disinfectants to remove infectious microorganisms from the hospital environment, particularly in the intensive care unit and other areas that house severely ill or immunocompromised patients. However, given that the mechanisms of action between antibiotic resistance and disinfection resistance may be similar, it is highly plausible (and even unavoidable) that drug-resistant bacteria can also develop a resistance to the biochemical disinfectants through alterations to porins, efflux mechanisms, and oxidative stress regulators (OxyR and PerR) [7].

Alternatives to chemical disinfectants can be used by hospitals to efficiently sterilize their surroundings. These include “no-touch” disinfection techniques like hydrogen peroxide vapor systems and ultraviolet (UV) radiation. Pathogens' ability to live on surfaces is decreased by UV light, especially UV-C irradiation, which effectively breaks down their DNA. Similarly, even in difficult-to-reach places, hydrogen peroxide systems emit a vapor that permeates and gets rid of microbiological contaminants. When utilized for terminal room disinfection, both techniques have been demonstrated to dramatically lower infection rates in healthcare institutions and healthcare-associated infections including MRSA and *C. difficile*. These strategies enhance general hygiene in healthcare environments by supplementing conventional cleaning techniques. UV light, at certain wavelengths, breaks the chemical links in DNA, killing the organism [8].

Inadequate disinfection may result in inefficient prevention and control of infectious diseases. If disinfection resistance does arise and goes unnoticed by healthcare providers, eventually it would have major repercussions including outbreaks of various serious infections. Therefore, it is an important issue that remains to be addressed in each individual healthcare facility as to whether the current sterilization and disinfection protocols can fully eliminate the infectious microbes present in the hospital environment [9]. There are other variables that contribute to bacterial resistance besides disinfectants. Inherent resistance mechanisms of microorganisms with extrinsic factors such as protective layers which either block or decrease the effects of antimicrobials, and intrinsic microbial traits, such as waxy walls in mycobacteria, and genetic resistant [10].

In the food processing sector, QACs are frequently utilized in disinfection procedures. It has been hypothesized that using them could result in the occurrence of resistant isolates or that susceptible isolates could develop resistance [11]. Gram-negative bacteria are generally more resistant to QACs than gram-positive bacteria. *Pseudomonas* spp. exhibit higher inherent resistance among gram-negative bacteria [11]. Ramzi et al.'s study demonstrated how some disinfectants based on quaternary ammonium affected a number of bacterial isolates, including those that were resistant to and sensitive to antibiotics. According to the test results, the three disinfectants are ineffective against certain isolated hospital–environmental isolates. The disinfectant spray inhibited *E. coli*, *S. aureus*, *E. coli*, and *P. aeruginosa* at 4 mg/mL [12]. Another study employed disinfectants (Deconex, Steranios, and frozen queinoa) on instruments and surfaces to combat *B. cepacia* and *E. faecalis*. Using nonlethal doses (sub–minimum inhibitory concentration (MIC)) of disinfectants does not kill germs, but instead builds their resistance [13]. Soto et al.'s study showed that a vinegar and hydrogen peroxide mixture can successfully eradicate *Candida albicans* and *S. aureus*. However, when the vinegar and hydrogen peroxide solution were used independently, microorganism counts decreased but were not successfully removed from the acrylic resin biofilms [14]. The study by Halstead et al. focused on acetic acid's widely applicable, accessible, and affordable qualities. In a more recent investigation, biofilm development in burn wounds was treated with a low dose of acetic acid (0.16–0.31%) [15].

Acetic acid is a well-known home remedy for cleaning. It has proven benefits for treating *Pseudomonas* infections in open wounds [16]. Due to its natural breakdown in the body or environment, acetic acid is not considered harmful or irritating. From a safety and technical perspective, acetic acid is inexpensive, widely accessible, efficient, and ease of use [17].

## 2. Materials and Methods

### 2.1. Sample Collection and Techniques

A total of 120 samples were taken from high-touch surfaces in a specialized patient area of Gulab Devi Hospital, Lahore, Pakistan. The Medical, Surgical, Obstetrics, and Gynecology wards, as well as the operating room, were the locations from where the samples were collected, that is, 24 samples per ward. In a specialized patient area, including tray tables, side tables, bed frames, IV poles, attendant seats, and bed handles, four samples were taken from each of the surfaces. These surfaces are highly exposed and touch frequently.

Samples were collected carefully using a sterile swab stick moistened with (0.9% *w*/*v*) physiological saline with linear swabbing for one minute applied to the surfaces of tray tables, side tables, bed frames, IV poles, attendant seats, and bed handles and then inserted into Amies transport medium (Thermo Fisher Scientific, TS0001A). They were transported within 30 min after labeling them properly to the microbiology laboratory. The choice of sampling method was based on standard microbiological techniques and following the previous study [18].

### 2.2. Isolation and Identification of Bacteria

The practical shelves were cleaned with 70% alcohol swabs before starting bench work. The samples were inoculated immediately on sheep blood agar and MacConkey agar plates (Appendix 1). Inoculated plates were incubated at 37°C for 18–24 h. After incubation, a growth pattern was observed on agar plates and isolated colonies proceeded for identification, whereas others were subcultured to obtain pure growth. After pure growth, microscopy such as gram stain was done to differentiate the gram-positive and gram-negative bacteria. Phenotypic methods have been used for the identification of bacteria. Isolates were identified by colony morphology and microscopic features. For confirmation, Analytical Profile Index (API) 20E was used for Enterobacteriaceae and biochemical tests (e.g., catalase, coagulase, DNAse, oxidase, triple sugar iron, motility, urease, citrate, indole, methyl red, and Voges–Proskauer) were used for other gram-negative bacteria. Cefoxitin (30 μg) disc was used to identify MRSA according to Clinical and Laboratory Standards Institute (CLSI) M100 [19]. *E. coli* (ATCC 25922) was used as a reference strain.

### 2.3. Determination of Antibacterial Activity of Disinfectants

The antibacterial activity of acetic acid and three other disinfectants such as phenol, sodium hypochlorite, and didecyldimethylammonium chloride (DDAC) was checked using broth microdilution method against all bacterial isolates isolated from hospital high-touch surfaces.

Acetic acid ≥ 99% (Sigma-Aldrich) was diluted to 6.9% by adding 6.9 mL of pure glacial acetic acid to 93.1 mL of distilled water. Working concentrations of acetic acid that were used in serial dilution are 6.9%, 3.4%, 1.7%, 0.86%, 0.43%, 0.21%, 0.10%, 0.05%, and 0.025%. Phenol was diluted to a 1% solution. Sodium hypochlorite was diluted to 0.1% by adding 0.1 mL of NaOCl to 99.9 mL of distilled water. Sodium hypochlorite and phenol were used in concentrations of 0.1% and 01%, respectively, as recommended by WHO and CDC [20, 21]. Working concentration of phenol was from a maximum of 01 μL/mL to a lowest of 0.0039 μL/mL. Working concentration of sodium hypochlorite was from 0.1 to 0.00039 μL/mL. QAC (DDAC) was diluted to 8.5% by adding 8.5 g of the tested compound in 91.5 mL of distilled water [2]. Working concentration of DDAC was 8.5 to 0.033 μL/mL.

### 2.4. Standard Broth Microdilution for MIC

To evaluate the antibacterial effects of the compounds that were tested, the MIC was assessed using the broth microdilution method according to the method proposed by CLSI M07-A11 and the previous study [22, 23]. The standard broth microdilution method was used because it is a recommended method by the European Committee on Antimicrobial Susceptibility Testing (EUCUST) and CLSI, and it requires low sample with a high accuracy rate [24, 25].

Initially, 100 μL of Mueller–Hinton (MH) broth was added into each well of the 96-well microtiter plate except for sterility control. Then, 100 μL of acetic acid was added to the first well and serially diluted till Well No. 09, for example, 6.9, 3.4, 1.7, 0.86, 0.43, 0.21, 0.10, 0.05, and 0.025 μL/mL. Bacterial inoculum was adjusted to a final concentration of 5 × 10^5^ CFU/mL according to CLSI M100. In the last step, 10 μL of the adjusted inoculum was added to each well containing 100 μL of antimicrobial agent in the dilution series except for sterility and negative control. The same procedure was used for other disinfectants with different concentrations. For the phenol, the concentrations were 01, 0.5, 0.25, 0.125, 0.062, 0.031, 0.015, 0.007, and 0.0039 μL/mL. Concentrations for sodium hypochlorite were 0.1, 0.05, 0.025, 0.0125, 0.0062, 0.0031, 0.0015, 0.0007, and 0.00039 μL/mL. DDAC was used in concentrations of 8.5, 4.25, 2.12, 1.06, 0.53, 0.26, 0.13, 0.066, and 0.033 μL/mL.

Well containing broth without antimicrobial agent for each organism has been used as growth control (No. 10). Well containing only broth was used as negative control (No. 11), and well containing only normal saline was used as sterility control (No. 12) (Appendix 2). The whole process is repeated for the remaining compounds under study. Inoculated microdilution plates were incubated at 35°C ± 2°C for 18–24 h in the incubator within 15 min of adding the bacterial inoculum. MIC is defined as the minimum concentration of the tested compound preventing visible growth of the organism in the wells detected by the unaided eye. MIC of the acetic acid and three other disinfectants was determined in comparison with the amount of growth in the growth control wells (no antimicrobial agent). Bacterial growth was assessed by checking turbidity in the wells. Clear wells indicated no visible bacterial growth. For the experiment to be considered valid, acceptable growth in control well should be ≥ 2-mm button or definite turbidity) [22].

### 2.5. Statistical Analysis

All data were validated, entered, and stored in a Microsoft Excel spreadsheet. Data analysis was done using Statistical Package for the Social Sciences (SPSS) version 22.0. ANOVA test was used for comparison of acetic acid with three disinfectants against different bacterial species. The ANOVA test was used because it allows for the comparison of means among multiple groups. Post hoc test was used to identify significant differences between disinfectants. A *p* value ≤ 0.05 has been considered statistically significant.

## 3. Results

A total of 140 bacterial isolates were isolated from 120 samples taken from different surfaces of hospital wards. The highest number of bacteria, 37 (26.42%), was found in the intensive care unit while the least counts, 31 (22.14%), were noted in general operation theater (OT). Out of these 120 surface samples, 28 (23.33%) did not show any growth.

### 3.1. Distribution of Bacteria Among Different Wards of Hospital

According to this study, a total of 76 (45.2%) *Staphylococcus aureus* isolates were isolated from 120 surface samples taken from different wards. Among 76 *S. aureus*, 13 bacterial isolates were resistant to methicillin. *S. marcescens* and *Klebsiella aerogenes* are among the least common bacteria that are found on inanimate surfaces of hospitals. Surgical ward had the most *Staphylococcus aureus* isolates (51.5%), followed by medical (48.6%), the intensive care unit (45.9%), and obstetrics and gynecology (37.5%), and OT has lowest (41.9%) *S. aureus* isolates. *Pseudomonas aeruginosa* is the second most frequent pathogen after *Staphylococcus aureus* (7.7%) with its prevalence in surgical ward as shown in Table 1.

### 3.2. Distribution of Bacteria on High-Touch Surfaces in a Specialized Patient Area

Table 2 shows the detailed data about the dissemination of bacteria on various high-touch surfaces in a specialized patient area. The bed frames are among the most contaminated surfaces followed by side tables. The IV poles, attendant seats, and bed handles almost have the same number of bacteria isolated from them. The largest percentage of no growth was seen on tray tables in samples taken from them. As previously, the *S. aureus* isolates are the most common microbes present in abundance on bed frames followed by *P. aeruginosa* and *K. pneumoniae.* It also shows that *A. baumannii* is mostly present on IV poles carrying fluids, IV drug bottles, or blood product bags.

### 3.3. Antibacterial Activity of Acetic Acid and Other Disinfectants

#### 3.3.1. MIC of Acetic Acid and Three Other Disinfectants Against Gram-Positive Bacteria

Table 3 shows the MIC value of acetic acid and three other disinfectants (phenol, sodium hypochlorite, and DDAC) against both gram-positive and gram-negative bacteria isolated from hospital high-touch surfaces. For acetic acid and the other three disinfectants, the MIC was found to vary in relation to the type of bacteria.

**Figure 1:**
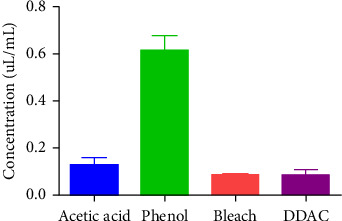
MIC of acetic acid and three other disinfectants against MRSA. ns means nonsignificant with *p* value ≥ 0.05 and ⁣^∗∗∗^ means significance with *p* value < 0.0001. Mean MIC of acetic acid was 0.12 ± 0.3 μL/mL, phenol 0.61 ± 0.6 μL/mL, sodium hypochlorite 0.08 ± 0.006 μL/mL, and DDAC 0.08 ± 0.02 μL/mL against MRSA.

The mean MIC of acetic acid was 0.14 ± 0.01 μL/mL against *S. aureus* which is lower than the phenol (0.78 ± 0.02 μL/mL), while sodium hypochlorite and DDAC inhibit the *S. aureus* at a concentration of 0.07 ± 0.00 μL/mL. Acetic acid shows excellent effectiveness against coagulase-negative *Staphylococcus* (CONS) with a mean MIC of 0.16 ± 0.02 μL/mL as compared to phenol 0.81 ± 0.09 μL/mL, while having comparable results as of sodium hypochlorite 0.06 ± 0.00 μL/mL. Acetic acid inhibited the *Enterococcus species* with low concentration (mean MIC = 0.08 ± 0.01 μL/mL) than the other three disinfectants including phenol (0.70 ± 0.12 μL/mL), sodium hypochlorite (0.09 ± 0.01 μL/mL), and DDAC (0.09 ± 0.01 μL/mL). Acetic acid showed growth inhibition of MRSA with mean MIC value of 0.12 μL/mL. Phenol had mean MIC value of 0.61 μL/mL against MRSA. The other two disinfectants, that is, bleach and DDAC, possessed inhibitory potential against MRSA with mean MIC value of 0.08 μL/mL as shown in Figure 1.

#### 3.3.2. MIC of Acetic Acid and Three Other Disinfectants Against Gram-Negative Bacteria

The mean MIC of acetic acid and other disinfectants including phenol, sodium hypochlorite, and DDAC against gram-negative bacteria is mentioned in Table 3.

For *K. pneumoniae*, the mean MIC of acetic acid was 0.09 ± 0.02 μL/mL which is lower than the phenol (0.75 ± 0.09 μL/mL) and DDAC (0.11 ± 0.03 μL/mL) while little higher than sodium hypochlorite (0.08 ± 0.00 μL/mL). The mean MIC of acetic acid was 0.13 ± 0.02 μL/mL against *P. aeruginosa* while phenol showed its growth inhibition for *P. aeruginosa* at concentration of 0.65 ± 0.08 μL/mL. The other two disinfectants, that is, sodium hypochlorite and DDAC, inhibit *P. aeruginosa* at 0.07 μL/mL. Acetic acid in our study showed the same results as sodium hypochlorite and DDAC against *A. baumannii* (MIC = 0.08 ± 0.02 μL/mL), while the efficacy of phenol is lower than these three agents with higher MIC value (0.92 ± 0.07 μL/mL).

Acetic acid was found to be better than all other three disinfectants for control of *S. marcescens* and *K. aerogenes* with mean MIC of 0.09 ± 0.04 and 0.05 ± 0 μL/mL, respectively. The acetic acid showed statistically significant results (*p* value ≤ 0.05) as compared to the other three disinfectants. DDAC also showed statistically significant results toward tested bacteria (*p* value ≤ 0.05). The other two disinfectants, phenol and sodium hypochlorite, showed nonsignificant results with *p* values ≥ 0.05.

## 4. Discussion

Hospital environments are most easily contaminated by pathogenic bacteria, which makes them ideal for the transmission of diseases and subsequent development of HAIs. High-touch surfaces in hospitals could spread nosocomial infections, which raises the risk of transmission [26, 27]. Despite rigorous sanitization procedures, the hospital setting is a significant source and spreader of bacteria that are resistant to antibiotics. Here, in this study, we aim to isolate bacteria present on hospital high-touch surfaces in a specialized patient area and to determine the efficacy of acetic acid in comparison with three other disinfectants including QAC, sodium hypochlorite, and phenol.

In our study, the culture result showed that 76 (45.2%) of bacterial isolates were *Staphylococcus aureus* isolated from different surfaces of various hospitals in patient areas. A similar study in a teaching hospital in Ethiopia reported mostly gram-positive isolates specifically *S. aureus* 63 (34.4%) from hospital surfaces [28]. A study by Hu et al. [29] declared that 50% of hospital surfaces are contaminated by *S. aureus,* which is likely to be shedding from the hands of healthcare workers and patients. The dominance of gram-positive bacteria on the hospital surfaces could be explained by the fact that these bacteria have a natural ability to persist on hospital surfaces from days to months [28, 30]. *S. aureus* is also a component of the human natural flora, which patients and medical staff frequently shed into the hospital environment, where they continue to stay viable [31, 32]. These bacteria would likely colonize and infect people getting medical care because they were resistant to ordinary disinfection techniques and spread readily in the surroundings. Additionally, the presence of *S. aureus* is a sign of poor clinical surface hygiene [28]. In this study, no growth was noted for 28 samples, the fact that 28 samples showed no growth suggests that the environment is clean and well-maintained. Notably, samples were collected the morning before the daily cleaning routine, and the last disinfection was carried out the day before. This demonstrates the hospital's commitment to appropriate hygiene standards and implies that the cleaning procedures in place are successful in preserving low bacteria levels for a prolonged amount of time.

We found both gram-positive and gram-negative bacteria on hospital high-touch surfaces such as tray tables, side tables, bed frames, IV poles, attendant seats, and bed handles. Among these, the bed frames were the most contaminated ones with the majority of *S. aureus* presence followed by *P. aeruginosa* and *K. pneumoniae*. Our study results concur with previous studies which reported a similar bacterial population [33, 34]. Enterobacteria like *Klebsiella pneumoniae* isolation strongly suggests fecal contamination likely secondary to poor personal hygiene and hand-washing practices among patients and healthcare professionals [35]. Since most researchers have reported that medical staff's hands are the most likely mode of transfer, it is possible that surfaces (like bed frames) that come into contact with hands most frequently while treating and transporting patients in any hospital setting have higher bacterial contamination levels than other surfaces [33, 36]. In this study, 17.1% *S. aureus* isolates were identified MRSA. The hospital environment such as high-touch surfaces is a major source of MRSA [37]. MRSA contamination in the hospital environment suggests that the hospital environment may be a significant source of nosocomial pathogens [38]. Hospital surface contamination by MDR bacteria poses a serious risk to public health. Hospitals must strictly adhere to the most recent standards and recommendations for hand hygiene, surface cleaning, and disinfection due to the existence of environmental bacterial reservoirs [34].

Use of disinfectants is critical for the control of infectious diseases. With the increasing number of publications on MDR bacteria, there has been a grave concern about the development of disinfectant resistance, particularly antibiotic cross-resistance [39]. Bacterial resistance to disinfectants based on phenol, chlorine, and alcohol compounds has been documented in the literature [2, 40]. The use of proper disinfectant agents is critical in good hospital practice, because inadequate disinfection may raise the risk of bacterial transmission and nosocomial infection. Therefore, this study investigates the disinfectant quality of acetic acid in comparison with three common disinfectants against bacteria isolated from hospital high-touch surfaces.

This study demonstrated a good inhibitory effect of acetic acid against *S. aureus* (0.14 μL/mL) and CONS (0.16 μL/mL), whereas a mean concentration of 0.08 μL/mL inhibits *Enterococcus species*. A study by Fraise et al. reported the efficacy of acetic acid against the methicillin-susceptible isolates of *S. aureus* (MSSA) (MIC: 0.625%) and MRSA (MIC: 0.625%) [41], but their study was limited only to air samples while we focus on samples from high-touch surfaces. Acetic acid also had antibacterial potential against mastitis-related pathogens with MIC of 0.125% against *S. aureus* [42]. The majority of bacteria are killed by acetic acid at concentrations as low as 0.5%. This toxic effect is primarily the result of acetic acid dissociation within microbial cells, which among other effects leading to a drop in intracellular pH and metabolic disruption by the anion [43]. The results of MIC vary among gram-negative and gram-positive bacteria. This can be explained by the fact that gram-positive and gram-negative bacteria have different cell wall structures, with the latter being able to limit the diffusion of hydrophobic substances through its LPS envelope [44]. Additionally, it can also depend on bacterial resistance to biocide and biofilm formation.

We noted the effects of acetic acid as a disinfectant against gram-negative pathogens related to the enteric tract, for example, *K. pneumoniae, P. vulgaris, E. coli, and A. baumannii* with mean MIC ranging from 0.08 to 0.25 μL/mL. In line with this, a study found that acetic acid is a valuable alternative to other methods in decontamination of sink drains in concentrations of 0.86%–13.8% against carbapenemase-producing *Enterobacteriaceae* [42]. Another study documented the effect of acetic acid against biofilm-producing bacteria related to burn patients including *A. baumannii, E. coli, P. aeruginosa, P. mirabilis, E. cloacae,* and *K. pneumoniae* [15]. There are previous reports on antibacterial activity of acetic against food borne pathogens, that is, *E. coli* 0157:07 and *Shigella* species, and found it a suitable compound for inactivation of foodborne pathogens as well [5, 45]. Acetic acid is believed to have antibacterial effects on *Salmonella* species associated with food by inhibiting bacterial DNA synthesis and depleting bacterial cells' energy stores [46]. Comparatively, we used acetic acid at low concentration against multiple types of bacteria than the concentration reported in these studies.

MIC of acetic acid is found to be 0.13% against *P. aeruginosa* which is in accordance with the previous reports [47]. Metallo-beta-lactamase–producing *Pseudomonas aeruginosa* biofilm was sensitive to 0.75% acetic acid, and it has proved to be a useful and simple method to decontaminate hospital sink drains [16]. Bacterial biofilms have been shown to be extremely resistant to antimicrobial treatments. According to a study, acetic acid not only killed planktonic bacteria but also bacteria developing biofilms. Acetic acid is found in liquid state at room temperature and pressure. When dissolved in a liquid, the salt sodium diacetate (NaHAc2) produces acetic acid and is as effective at killing biofilm-forming bacteria [48].

In addition, acetic acid showed greater potential than all three other disinfectants against *S. marcescens* and *K. aerogenes*. Acetic acid and DDAC among the three disinfectants show statistically significant results. DDAC has significant results that may be due to the use of high concentrations (8.5%). DDAC has been found active against gram-positive bacteria only while the gram-negative bacteria are less susceptible due to intrinsic resistance [2, 49]. This study also found no effects of phenol and sodium hypochlorite against studied bacterial pathogens [2]. A study in mice models shows that quaternary ammonium disinfectants significantly decreased reproductive health with impaired fertility [50].

There is limited information about acetic acid in hospital practice, although one study compared the efficacy of a 4% acetic acid solution to chlorine tablet disinfectants for hospital surfaces. Acetic acid reduced CONS by 70.8%, reduced *K. pneumoniae* by 19.5%, and fully eradicated *Bacillus* species, among other results. Although chlorine pills were efficient against specific bacteria, they exceeded acetic acid for overall microbial reduction, including the removal of *K. pneumoniae* [51]. In another investigation, metallo-b-lactamase–producing *P. aeruginosa*-positive sinks were treated once a week with 24% acetic acid and cultured repeatedly. Acetic acid treatment of colonized sink drains on a weekly basis resulted in negative cultures and the termination of transmission [16].

Effective and economical disinfectants are needed to kill bacteria on the surfaces of hospitals and prevent the nosocomial infections. For that purpose, acetic acid has the potential to be a good alternative to available disinfecting agents. Acetic acid has less toxicity, inexpensive (2.5 L of 99% acetic acid costs < US $100), and easily available with strong bactericidal effects [52]. The acetic acid efficacy is not only limited to an antibacterial agent, but it is also recommended as a promising, easily administered, cheap, and well-tolerated adjunctive therapy against COVID-19 [53].

This study has some limitations. This study was done in a single center. Multicenter involvement would have helped identify more bacterial isolates and also the distribution pattern of bacteria in different hospitals. In this study, we used only clinical isolates and did not check their activity against ATCC bacterial strains. The antifungal activity against fungi is not done as well as minimum fungicidal activity (MFC) that is another study limitation. The complete resistance pattern of isolated bacteria was not investigated as it was beyond the scope of this study in terms of time and resources. Molecular characterization of resistant bacteria has not been investigated. Further studies are needed to confirm its mechanism of action and toxicity profile.

## 5. Conclusions

We concluded that hospital surfaces are contaminated by multiple gram-positive and gram-negative bacteria. It was found that the acetic acid is highly effective as compared to phenol and DDAC against bacteria isolated from hospital high-touch surfaces. Phenol was found nonsignificant while DDAC showed good results at high concentrations as compared to phenol. Comparatively sodium hypochlorite showed excellent efficacy against bacterial isolates. Acetic acid can be used as an effective surface disinfectant in the hospital settings. Owing to its low toxic profile, acetic acid can be a good, effective, and cheaper alternative to commonly available relatively toxic chemical disinfectants.

## Figures and Tables

**Table 1:** Distribution of bacteria among various wards at Gulab Devi Hospital, Lahore, Pakistan.

Bacterial isolates	Wards					Total *n* (%)
	Medical *n* (%)	ICU *n* (%)	Surgical *n* (%)	Obstetrics and gynecology *n* (%)	OT *n* (%)	
No growth	06 (17.1)	04 (10.8)	04 (12.1)	06 (18.8)	08 (25.8)	28 (16.7)
*S. aureus*	17 (48.6)	17 (45.9)	17 (51.5)	12 (37.5)	13 (41.9)	76 (45.2)
*K. pneumoniae*	03 (8.6)	02 (5.4)	01 (3.0)	01 (3.1)	01 (3.2)	08 (4.8)
*P. aeruginosa*	02 (5.7)	03 (8.1)	04 (12.1)	02 (6.2)	02 (6.5)	13 (7.7)
*P. vulgaris*	01 (2.9)	02 (5.4)	02 (6.1)	01 (3.1)	0 (0.0)	06 (3.6)
*E. coli*	02 (5.7)	01 (2.7)	01 (3.0)	03 (9.4)	0 (0.0)	07 (4.2)
*CONS*	01 (2.9)	01 (2.7)	01 (3.0)	03 (9.4)	02 (6.5)	08 (4.8)
*Enterococcus species*	01 (2.9)	02 (5.4)	01 (3.0)	01 (3.1)	0 (0.0)	05 (3.0)
*Bacillus species*	02 (5.7)	02 (5.4)	01 (3.0)	0 (0.0)	0 (0.0)	05 (3.0)
*A. baumannii*	0 (0.0)	02 (5.4)	01 (3.0)	01 (3.1)	03 (9.7)	07 (4.2)
*S. marcescens*	0 (0.0)	0 (0.0)	0 (0.0)	02 (6.2)	02 (6.5)	04 (2.4)
*K. aerogenes*	0 (0.0)	01 (2.7)	0 (0.0)	0 (0.0)	0 (0.0)	01 (0.6)
Total	35 (100)	37 (100)	33 (100)	32 (100)	31 (100)	168 (100)

**Table 2:** Distribution of bacteria on high-touch surfaces at Gulab Devi Hospital, Lahore, Pakistan.

Organisms isolated	Surfaces						Total
	Tray table	Side table	Bed frame	IV pole	Attendant seat	Bed handle	
No growth	8	3	2	6	3	6	28
	30.8%	10.7%	6.1%	22.2%	11.1%	22.2%	16.7%

*S. aureus*	12	13	16	13	11	11	76
	46.2%	46.4%	48.5%	48.1%	40.7%	40.7%	45.2%

*K. pneumoniae*	1	1	2	1	1	2	8
	3.8%	3.6%	6.1%	3.7%	3.7%	7.4%	4.8%

*P. aeruginosa*	1	4	5	0	1	2	13
	3.8%	14.3%	15.2%	0.0%	3.7%	7.4%	7.7%

*P. vulgaris*	1	2	1	1	0	1	6
	3.8%	7.1%	3.0%	3.7%	0.0%	3.7%	3.6%

*E. coli*	1	1	1	0	3	1	7
	3.8%	3.6%	3.0%	0.0%	11.1%	3.7%	4.2%

CONS	1	1	1	1	4	0	8
	3.8%	3.6%	3.0%	3.7%	14.8%	0.0%	4.8%

*Enterococcus species*	0	0	1	1	3	0	5
	0.0%	0.0%	3.0%	3.7%	11.1%	0.0%	3.0%

*Bacillus species*	0	2	0	1	0	2	5
	0.0%	7.1%	0.0%	3.7%	0.0%	7.4%	3.0%

*A. baumannii*	0	1	2	3	0	1	7
	0.0%	3.6%	6.1%	11.1%	0.0%	3.7%	4.2%

*S. marcescens*	1	0	1	0	1	1	4
	3.8%	0.0%	3.0%	0.0%	3.7%	3.7%	2.4%

*K. aerogenes*	0	0	1	0	0	0	1
	0.0%	0.0%	3.0%	0.0%	0.0%	0.0%	0.6%

Total	26	28	33	27	27	27	168
	100.0%	100.0%	100.0%	100.0%	100.0%	100.0%	100.0%

**Table 3:** MIC of acetic acid and three other disinfectants against isolated bacteria from high-touch surfaces.

Bacteria	Number of bacterial isolates	Acetic acid (μL/mL)	Phenol (μL/mL)	Sodium hypochlorite (μL/mL)	Didecyldimethylammonium ammonium chloride (μL/mL)
*S. aureus*	76	0.14 ± 0.01	0.78 ± 0.02	0.07 ± 0.00	0.07 ± 0.0
*K. pneumoniae*	08	0.09 ± 0.02	0.75 ± 0.09	0.08 ± 0.00	0.11 ± 0.03
*P. aeruginosa*	13	0.13 ± 0.02	0.65 ± 0.08	0.07 ± 0.00	0.07 ± 0.01
*P. vulgaris*	06	0.14 ± 0.03	0.83 ± 0.10	0.08 ± 0.01	0.16 ± 0.04
*E. coli*	07	0.25 ± 0.06	0.78 ± 0.10	0.09 ± 0.00	0.17 ± 0.04
CONS	08	0.16 ± 0.02	0.81 ± 0.09	0.06 ± 0.00	0.13 ± 0.04
*Enterococcus* species	05	0.08 ± 0.01	0.70 ± 0.12	0.09 ± 0.01	0.09 ± 0.04
*Bacillus* species	05	0.09 ± 0.03	0.60 ± 0.10	0.09 ± 0.01	0.07 ± 0.02
*A. baumannii*	07	0.08 ± 0.02	0.92 ± 0.07	0.08 ± 0.01	0.08 ± 0.03
*S. marcescens*	04	0.09 ± 0.04	0.87 ± 0.12	0.10 ± 0.0	0.11 ± 0.05
*K. aerogenes*	01	0.05 ± 0	0.50 ± 0.0	0.10 ± 0.0	0.06 ± 0.0

*Note:* Data are presented as mean ± standard error of mean.

## Data Availability

All the authors of the study had full access to all the data and had final responsibility for the decision to submit it for publication.

## Supporting Information

Appendix 1: Bacterial growth on blood agar plates, the isolated colonies showed growth of *S. aureus*.

Appendix 2: Figure of a 96-well microtiter plate for MIC.
