# Supplementary material for: Evaluation of the Antibacterial Activity of Acetic Acid in Comparison With Three Disinfectants Against Bacteria Isolated From Hospital High-Touch Surfaces
Source: Scientifica (Cairo). 2025 Mar 26;2025:7598027. doi: 10.1155/sci5/7598027 (PMC11964715; doi:10.1155/sci5/7598027)
Supplement: Supporting Information — Additional supporting information can be found online in the Supporting Information section. [file 7598027.f1.docx]

Supplementary file

**Appendix 1:** **Bacterial growth on blood agar plates.** the isolated colonies showed growth of S. aureus after 24 hour of incubation


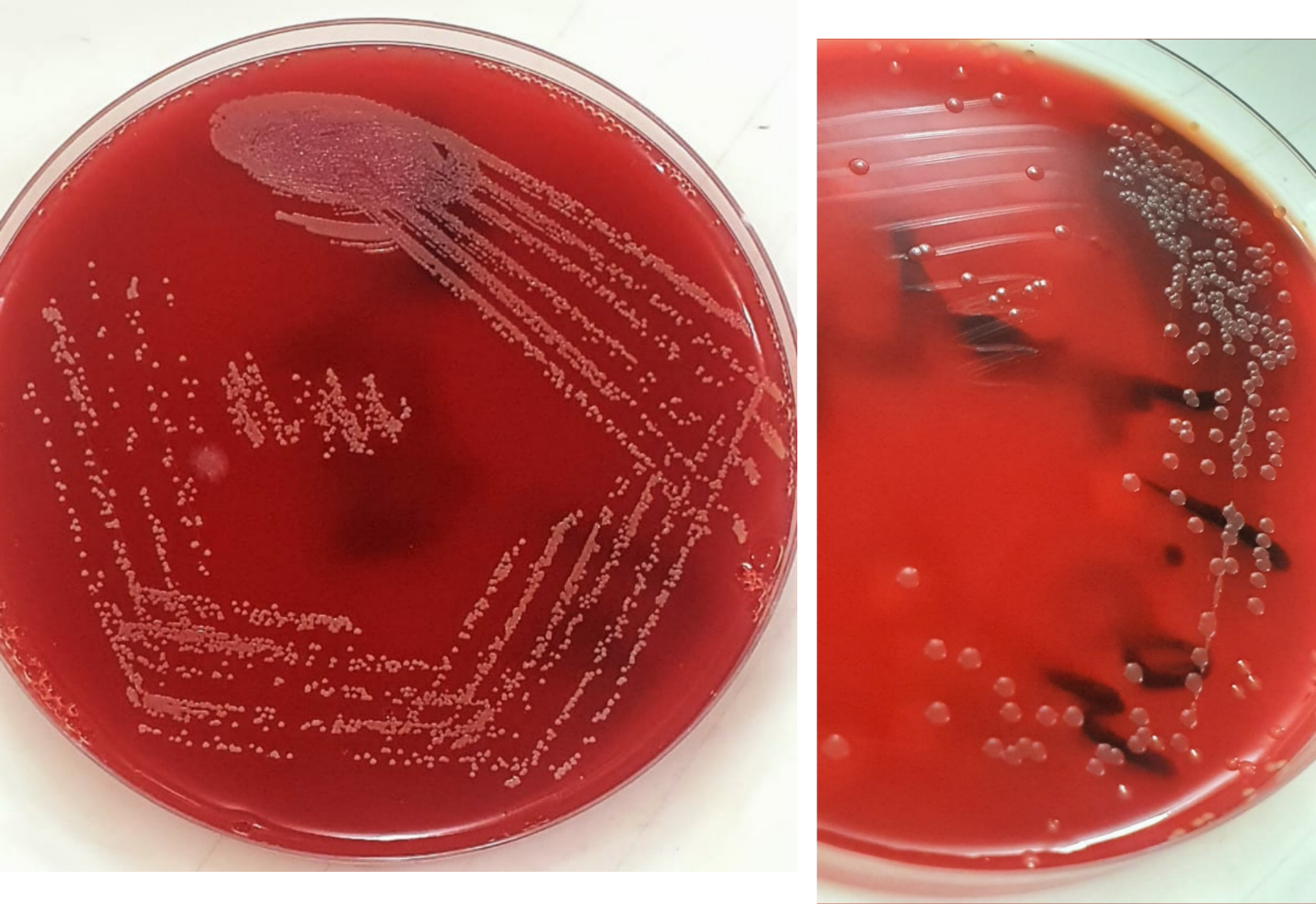


**Appendix 2: Figure of 96 well microtiter plate for MIC**


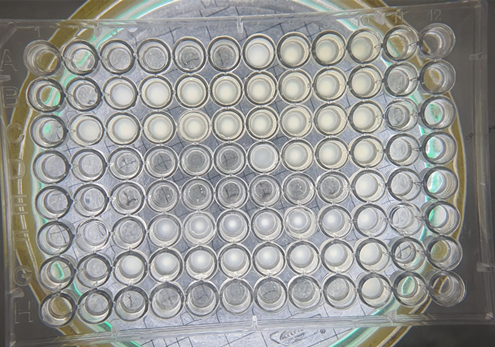


**Determination of Minimal inhibitory concentration of acetic acid and other three disinfectants against isolated bacteria.** A, Acetic acid ; B, Phenol; C, Sodium Hypochlorite ; D, DDAC; E, Acetic acid ; F, Phenol; G, Sodium Hypochlorite ; H, DDAC. The number from 1 to 09 at each row of the microplate indicate different concentration from highest to lowest in the wells of acetic and other disinfectants as indicate above. Well number 10 at each column is growth control; well 11 is negative control only media; well 12 is sterility control (only normal saline).
